# The role of muscle-specific MicroRNAs in patients with chronic obstructive pulmonary disease and skeletal muscle dysfunction

**DOI:** 10.3389/fphys.2022.954364

**Published:** 2022-10-21

**Authors:** Hui Zhao, Peijun Li, Jihong Wang

**Affiliations:** ^1^ Department of Sports Rehabilitation, Shanghai University of Sport, Shanghai, China; ^2^ School of Physical Education, Shanghai University of Sport, Shanghai, China

**Keywords:** chronic obstructive pulmonary disease, MicroRNAs, skeletal muscle dysfunction, respiratory muscles, peripheral skeletal muscles

## Abstract

Skeletal muscle dysfunction is a systematic manifestation of chronic obstructive pulmonary disease (COPD), which is manifested through the changes in the respiratory and peripheral muscle fiber types, reducing muscle strength and endurance, and muscle atrophy. Muscle dysfunction limits the daily mobility, negatively affects the quality of life, and may increase the patient’s risk of mortality. MicroRNAs (miRNAs) as the regulators of gene expression, plays an important role in modulating skeletal muscle dysfunction in COPD by regulating skeletal muscle development (proliferation, differentiation), protein synthesis and degradation, inflammatory response, and metabolism. In particular, muscle-specific miRNAs (myomiRs) may play an important role in this process, although the different expression levels of myomiRs in COPD and skeletal muscle dysfunction and the mechanisms underlying their role remain unclear. In this paper, we review the differential expression of the myomiRs in COPD to identify myomiRs that play a role in skeletal muscle dysfunction in COPD. We further explore their possible mechanisms and action in order to provide new ideas for the prevention and treatment of the skeletal muscle dysfunction in COPD.

## 1 Introduction

Chronic obstructive pulmonary disease (COPD) is a very common clinical disease characterized by irreversible airflow limitation, insidious onset, and recurrent and progressive time course (Vogelmeier et al., 2017). Globally, there is an increasing yearly number of COPD cases, thus resulting in an increasing mortality rate (GBD 2015 Chronic Respiratory Disease Collaborators, 2017; Woo et al., 2019; Rehman et al., 2020). Previous research has found that COPD is not only highly injurious to the lungs but can also cause an extensive extrapulmonary effect, of which skeletal muscle dysfunction is a manifestation of extrapulmonary injury that occurs during the early stages of the disease. Loss of muscle mass and decreases in muscle strength manifest as the primary symptoms, affecting quality of life and accurately predicting mortality in COPD patients (van Helvoort et al., 2007). Various factors and mechanisms are involved in COPD muscle dysfunction, such as oxidative stress, systemic inflammation, mitochondrial disorders, and autophagy (Puente-Maestu et al., 2011; Fermoselle et al., 2012; Gea et al., 2013). Studies have found that the epigenetic events are also potential modifiers of muscle mass and its function in COPD patients (Lewis et al., 2012; Barreiro and Sznajder, 2013; Donaldson et al., 2013). Among them, microRNAs (miRNAs) are non-coding single-stranded RNA molecules (18–24 nucleotides) that play important roles in cell proliferation, differentiation, apoptosis, disease onset, and progression by binding to the translation region (3′ UTR) of target mRNAs and inducing mRNA degradation or inhibition of translation (Liu et al., 2015; Zhang et al., 2015). They are also considered as small molecules with high potential to target diagnostics and therapeutics (Deiters, 2010), and studies have shown that miRNAs play a key role in regulating skeletal muscle cell proliferation, differentiation, apoptosis, and senescence (McGregor et al., 2014; Mytidou et al., 2021).Muscle-specific miRNAs (myomiRs), such as miR-1, miR-206, miR-133, miR-208, and miR-499, may also exhibit dysregulated expression and cause the development of skeletal muscle dysfunction in COPD (Wang et al., 2018).

## 2 COPD skeletal muscle dysfunction

Studies have confirmed the prevalence of skeletal muscle dysfunction in patients with COPD, which can occur during any period of the disease (GOLD I to IV) and mainly in respiratory and peripheral skeletal muscles including the diaphragm and quadriceps, respectively (Seymour et al., 2010; Gea et al., 2015; Barreiro, 2017). More specifically, in COPD patients the respiratory muscles exhibit increased endurance and strength for a short period of time, while peripheral skeletal muscles exhibit decreased endurance and strength, showing a clear difference between the two types in terms of skeletal muscle functional changes. However, both lead to exacerbation of the patient’s condition and increases the length of hospitalization and risk to mortality. Moreover, most studies have shown that skeletal muscle dysfunction can occur early in respiratory dysfunction (Coronell et al., 2004; Gea et al., 2013; Barreiro and Gea, 2015), making it important to investigate the mechanisms of the onset and development of skeletal muscle dysfunction in patients with COPD.

### 2.1 Respiratory muscle dysfunction

It is known that the respiratory muscles are crucial to the respiratory system, particularly the diaphragm. It has been established that respiratory muscle dysfunction may cause hypercapnic respiratory failure, limited exercise capacity, increased risk of acute exacerbations, and eventual death in COPD patients (Vilaró et al., 2010). COPD results in alterations to the function of the diaphragm because of its abnormal breathing pattern and frequency, causing diaphragm dysfunction (Zanforlin et al., 2015).In addition, some studies have found that the main features of the diaphragm in terms of skeletal muscle structure are its shortened muscle segments, higher percentage of type II to type I fibers, increased numbers of capillaries. And increased mitochondrial density compared with the healthy groups (Stubbings et al., 2008; Levine et al., 2013).These all suggest adaptive changes and better fatigue tolerance in the COPD diaphragm. Moreover, another study found similar adaptive changes in the diaphragm, suggesting that the functions of the diaphragm in COPD patients undergo positive adaptations similar to the training effect because of increasing ventilatory load, making them more resistant to fatigue (Doucet et al., 2004). However, as the disease progresses and worsens, COPD patients experience severe hypoxia and the diaphragm shows ganglion rupture and more lactic acid production from the mitochondrial hypoxia, leading to diaphragm dysfunction (Orozco-Levi et al., 2001; Clanton and Levine, 2009; Klimathianaki et al., 2011). Because of the above characteristics, diaphragm dysfunction is not detected early, and by the time it is detected patients are usually in the moderate and higher stages of the disease (Ferguson et al., 1990). These prove detrimental to the treatment and leads to poor exercise tolerance, making it crucial to focus on respiratory muscle dysfunction during pulmonary rehabilitation (Gea et al., 2013).

### 2.2 Peripheral skeletal muscle dysfunction

Peripheral skeletal muscle dysfunction in COPD patients is impaired in all extremities, and is mainly manifested through the changes in the muscle cross-sectional area and fiber type. This results in reductions in motor strength, endurance, and resistance to fatigue. Studies have indeed confirmed that COPD patients with concomitant skeletal muscle dysfunction is not directly related to the degree of their airway obstruction, and even in the early stages of the disease, muscle function of the extremities decrease significantly, especially in the lower extremities (Man et al., 2003; Seymour et al., 2010). A study of a large United Kingdom and Dutch COPD cohort showed that the proportion of patients with early COPD who developed skeletal muscle weakness was 28% higher compared to healthy controls (Seymour et al., 2010).Specifically, deficits included reductions in the quadriceps cross-sectional area and volume, thus decreasing muscle strength and motility, but no difference in muscle fiber type (Puig-Vilanova et al., 2014b). Patients with advanced COPD also show a shift in fiber type of quadriceps (from type I to type II fiber phenotype), with type II fibers showing lower calcium sensitivity relative to type I fibers. These are predominantly anaerobically metabolized and more fatigable, thus producing less maximal force (Stephenson and Williams, 1981).In addition, patients with advanced COPD tend to have reduced type II fiber cross-sectional area and loss of muscle mass, leading to a decrease in muscle strength, muscle endurance levels, and fatigue resistance (Gea et al., 2013)and skeletal muscle dysfunction (Stephenson and Williams, 1981; Maltais et al., 2014; Barreiro and Jaitovich, 2018). Moreover, COPD patients with altered fiber types had short 6-min walking distances and poor exercise endurance (Natanek et al., 2013). For instance, Maltais et al. (2014) found that in patients with severe COPD, the cross-sectional area and maximum muscle strength of the quadriceps were reduced by roughly 30% compared to healthy people of the same age. In another study, COPD patients in all GOLD stages showed decreased cross-sectional area and strength in the quadriceps compared to controls. Patients in GOLD stage I also showed a 17% decrease in muscle cross-sectional area (Shrikrishna et al., 2012). In addition, abnormalities in the muscle metabolic status, such as reduced capillaries and reduced levels of mitochondrial oxidative metabolic enzymes, also contribute to the skeletal muscle dysfunction (Zhu et al., 2015). Gosker et al. observed not only a decrease in the proportion of type I fibers and an increase in the proportion of type II fibers of the lateral femoral muscles of COPD patients, but also a decrease in the number of myofibrillar mitochondria and in the content of oxidative enzymes, thus leading to reduced endurance and fatigue resistance in the muscles of COPD patients (Gosker et al., 2007). In animal studies, reduced skeletal muscle type I fiber size and ratio, downregulated mitochondrial activity, and increasing the levels of oxidative stress in the experimental animal models of chronic cigarette smoke exposure have been found to be the cause of skeletal muscle dysfunction in COPD (Barreiro and Jaitovich, 2018).

## 3 Differential MyomiR expression in COPD compared to healthy controls

As a regulator of gene expression, miRNAs plays an integral role in the development of cell growth and death, and are involved in the regulation of multiple physiological functions in the skeletal muscle cell proliferation, differentiation, skeletal muscle remodeling, and vascular regeneration (Dai et al., 2016; Horak et al., 2016). Studies have shown that dysregulated miRNAs are closely associated with the onset and progression of COPD disease (Conickx et al., 2017; Musri et al., 2018). The myomiRs are associated with muscle phenotypic regulators and are involved in regulating the different stages of muscle growth and development (O'Rourke et al., 2007; van Rooij et al., 2009; Novák et al., 2013).Abnormal expression patterns of myomiRs in the skeletal muscle of COPD patients have also been found, thus being associated with the skeletal muscle dysfunction and respiratory function in COPD.

### 3.1 MyomiR expression in respiratory muscles of COPD patients

Puig et al. noted that myomiRs were expressed differentially in the respiratory muscles in patients with COPD compared to age-matched sedentary controls. miR-1, miR-133, and −206 were downregulated in the diaphragms of mild, moderate and severe COPD patients, with miR-206 being more downregulated, whilst histone deacetylase 4 (HDAC4) and myocyte enhancer factor 2 (MEF2) protein levels were increased, muscle fibre type and cross-sectional area size does not differ between patients with mild COPD and healthy sedentary controls, whilst an increase in the proportion of diaphragm fibre type I was found in moderate and severe COPD patients with no difference in their cross-sectional area. In addition, this specific pattern of miRNA expression may be due to the continuous exposure of the respiratory muscles to inspiratory load. This study also found that, although total muscle strength of respiratory muscles of COPD patients was not reduced, diaphragm strength and exercise tolerance were reduced compared to the healthy sedentary controls (Puig-Vilanova et al., 2014a).

### 3.2 MyomiR expression in peripheral skeletal muscles of COPD patients

Puig-Vilanova et al. (2014b), further examined COPD patients compared to healthy controls and found high levels of miR-1 expression in the lateral femoral muscles of mild COPD patients, including positive correlations with patients’ quadriceps maximal velocity contraction (QMVC) and forced expiratory volume in one second (FEV1). MiR-133 expression and HDAC4 expression levels were also elevated, while no significant differences in the expression of other miRNAs (miR-206, −486, −27a, −29b, and −181a) were found. In comparing body composition of healthy controls and COPD patients, the researchers also found that body composition (e.g., muscle, bone, fat, water, and minerals, which are here mainly body mass index [BMI] and fat-free mass index [FFMI] measurements) is preserved in patients with mild COPD (Puig-Vilanova et al., 2014b). It was hypothesized that myomiR expression upregulated may operate in a network to ensure continuous muscle repair process after an injury, with positive regulation of skeletal muscle function. It has been also shown that high miR-1 expression has an inhibitory effect on HDAC4 and promotes myoblast differentiation and repairs muscle damage (Saccone et al., 2014). In another experiment by Puig-Vilanova et al. (2015), compared to healthy sedentary controls, the expression of miR-1, miR-206, and miR-27a in the lateral femoral muscle, and the levels of lysine acetylated protein, histone, acetylated histone 3, serum response factor (SRF), and follicle inhibitors were found to be elevated in all patients with COPD, while the expression of miR-133, histone deacetylase 3, HDAC4, and insulin-like growth factor-1 (IGF-1) decreased. There was also a significant negative correlation between miR-206 expression level and QMVC. In contrast, A study by Lewis et al. (2012) reported that miR-1 expression levels were significantly reduced in the lateral femoral muscle and elevated in HDAC4 and IGF-1 in patients with severe COPD. MiR-1 was positively correlated with FEV1 and negatively correlated with protein kinase B (Akt/PKB) phosphorylation, and its expression may be correlated with several factors such as smoking history, lung function, FFMI, 6-min walking distance, and type I fiber percentage; miR-133 and miR-206 expression levels were the same between patients and healthy controls in the lateral femoral muscle, and their expressions were also found to be negatively correlated with daily physical activity in severe patients (Lewis et al., 2012). Finally, the expression of miR-1 in peripheral skeletal muscle was found to differ with different degrees of the disease, which may be due to the muscle-specific expression of miR-1 (Chen et al., 2006; Luo et al., 2013). However, research by Puig et al. has shown that miR-1 expression levels were found to increase in the lateral femoral muscles of COPD patients with severe muscle weakness, thus contradicting the findings of Lewis, which may be partly due to reduced expression levels of HDAC4. It has also been shown that the miR-1 and HDACs have mutual regulatory roles. Specifically, inhibiting the HDACs induces upregulation of the miR-1 expression in mouse muscle regeneration models (Saccone et al., 2014).

### 3.3 Circulating MyomiR expression in blood of COPD patients

MiRNAs circulate in the blood, are resistant to endogenous RNases, and can be stably present in the blood, thus making it possible to study circulating miRNA (Mitchell et al., 2008). In comparing the miRNA profiles of patients with COPD (*n* = 103) to healthy controls (*n* = 25), plasma levels of miR-1, miR-499, miR-133 and miR-206 were all found to be higher in severe COPD patients (Donaldson et al., 2013). Significantly higher expression levels of miR-1 and miR-499 were also found in COPD patients compared to controls (2.5-fold and 1.5-fold, respectively). Moreover, plasma miR-1 levels were negatively correlated with FEV1 and FFMI, and patients with GOLD IV COPD had lower plasma miR-499 and miR-206 than those with GOLD II COPD. Collectively, these significantly higher plasma myomiRs in COPD patients compared to controls suggest that this difference is associated with an increase in fiber type shifts and muscle atrophy (Donaldson et al., 2013). In addition, plasma levels of myomiRs were found to increase with severity of muscle atrophy in COPD patients. However, as the severity of muscle atrophy in COPD patients continues to increase, plasma levels of skeletal muscle-specific miRNAs appear to decrease (Whittom et al., 1998; Donaldson et al., 2013). Many studies suggest that circulating miRNAs may be promising biomarkers for early disease detection, prognosis, or development of new therapeutic targets (Leidinger et al., 2011; Sanfiorenzo et al., 2013). Associated findings showed that miR-206 has a long half-life in the blood, is stable, and has ideal biomarker criteria, such as being measurable with minimal invasiveness, high specificity, and high sensitivity, all of which allow for rapid and accurate detection. Studies have also shown that increased circulating miR-206 levels are associated with the outcome of muscle fiber type transformation and increases with muscle wasting in patients with COPD, suggesting that miR-206 could be a biomarker in monitoring muscle dysfunction (Donaldson et al., 2013; Ulivi and Zoli, 2014).

As Table 1 shows, myomiRs have been found to be expressed abnormally in both the respiratory and peripheral muscles of COPD patients (Table 1). The distribution of results shows that expression patterns were different between the respiratory and peripheral muscles, with the miRNAs showing a decreasing trend in respiratory muscles and an increasing trend in peripheral muscles. A large number of studies have found that the expression of specific miRNAs in muscles alters during exercise and body-loading (Nielsen et al., 2010; Davidsen et al., 2011). Circulating myomiRs initially are elevated in COPD patients and subsequently decrease with disease progression, a phenomenon that suggests severe skeletal muscle atrophy in COPD.

**TABLE 1:** Differential myomiR expression in COPD compared to healthy controls.

Study	Group	Sample size	Degree	Tissue	miRNAs(Targets)	Lung function	Skeletal muscle function
Puig-Vilanova et al. (2014a)	CGHG	1810	Mild Moderate, severe	Diaphragm	miR-1↓, miR-133↓(SRF↑), miR -206↓, miR-486, miR -27a, miR-29b, and miR -181a	FEV1 (%pred)↓, FEV1/FVC↓	Exercise endurance↓, Diaphragm strength↓
Puig-Vilanova et al. (2014b)	CGHG	1313	Mild	Lateral femoral muscle	miR-1↑ (HDAC4↑), miR-133↑, miR -206, miR -208, and miR -499	miR-1 was positively correlated with FEV1	miR-1 was positively correlated with QMVC
Puig-Vilanova et al. (2015)	CGHG	4119	Severe	Lateral femoral muscle	miR-1↑(HDAC4↓, IGF-1↓), miR-206↑, miR-133↓(SRF↑), miR-27a↑, miR-486, and miR-29b		miR-206 was significantly and negatively correlated with QMVC
Lewis et al. (2012)	CGHG	3114	Severe	Lateral femoral muscle	miR-1↓(HDAC4↑, IGF-1↑, MEF2↓, SRF↓), miR-133, and miR-206	miR-1 was positively correlated with FEV1	Expression of miR-133 and miR-206 was negatively correlated with daily physical activity in severe COPD patients
Donaldson et al. (2013)	CGHG	10325	Mild Moderate, severe	Blood plasma	miR-1↑(HDAC4↑), miR-206↑, miR-133 and miR-499↑	miR-1 was negatively correlated with FEV1 and FFMI	miR-133, −206 and −499 were positively correlated with NF-κBp50 in quadriceps

Abbreviations: CG:COPD, group; COPD, chronic obstructive pulmonary disease; FFMI, fat-free mass index: FEV1, forced expiratory volume in one second; FEV1%pred, expected value of exertional expiratory volume in the first second; FVC, forced vital capacity; HDAC4, histone deacetylase 4; HG: healthy control group; IGF-1, insulin-like growth factor-1; MEF2, myocyte enhancer factor 2; NF-κBp50, enhanced p50 of κ-light chain of nuclear factor-activated B cells; QMVC, quadriceps muscle maximum contractility; SRF, serum response factor; down(↓); up (↑).

## 4 The role of MyomiRs on skeletal muscle dysfunction in COPD

Muscle-specific miR-1, miR-133 and miR-206 are the most studied and characterized miRNAs to date (Ma et al., 2019), showing varying expression levels across the respiratory and peripheral skeletal muscles in COPD. However, muscle-specific expression of these miRNAs are expressed differently in association with the skeletal muscle fiber type transformation or muscle atrophy in COPD(Lewis et al., 2012; Donaldson et al., 2013). These suggest that myomiRs regulate the internal changes of the skeletal muscle through multiple signal pathways during the state of COPD, thus affecting skeletal muscle function.

### 4.1 miR-1

#### 4.1.1 miR-1 affects the skeletal muscle protein synthesis and muscle growth inhibitor activity

Autophagy is a highly conserved self-protection mechanism preserved during evolution and plays an important role in maintaining the homeostasis of skeletal muscle protein metabolism, metabolic waste removal, structural reconstruction, and other cellular environmental homeostatic functions (Boya et al., 2013). It is also established that autophagy plays an important role in skeletal muscle atrophy in COPD patients (Guo et al., 2013). It is a tightly regulated process, and the phosphatidylinositol 3-kinase (PI3K)/Akt/mammalian target of rapamycin (m TOR) signaling pathway is an important component in regulation (Meijer and Codogno, 2006).IGF-1 deficiency and inhibition in the mTOR pathway can induce autophagy, which leads to much greater protein catabolism than anabolism through activation of the protein hydrolysis system, resulting in skeletal muscle loss and muscle atrophy (Sandri, 2008; Joassard et al., 2013; Sakuma et al., 2017). An increasing number of studies have confirmed that the IGF-1/PI3K/Akt pathway is regulated by various miRNAs. Among them, miR-1, which is highly expressed in the lateral femoral muscle of patients with severe COPD, targets and inhibits the expression of IGF-1, causing a decrease in IGF-1 levels in plasma and muscle and inducing autophagy to occur, thereby reducing the protein synthesis rate and causing a decrease in muscle mass (Sandri, 2008; Coşkun et al., 2009; Elia et al., 2009; Corbo et al., 2014). It has been shown that IGF-1 expression affects the expression level of miR-1, and the two have an interactive effect. (Tijsen et al., 2012). In addition, muscle growth inhibitor (myostain) and growth differentiation factor 15 (GDF-15) expressions were detected elevating in COPD (Patel et al., 2016; Zhang et al., 2022), thereby promoting muscle atrophy and inhibiting muscle expression of miR-1. A decrease in miR-1 increases the myostain activity, leading to a decrease in skeletal muscle myogenesis and a subsequent decrease in miR-1 in the lateral femoral muscle of COPD patients (Al-Mudares et al., 2020).

#### 4.1.2 miR-1 regulates the skeletal muscle fibre type expression and diaphragm overload adaptation

HDAC4 inhibits muscle differentiation and skeletal myogenic gene expression (Lu et al., 2000). As indicated by previous research (Lewis et al., 2012), severe COPD patients have exhibited a downregulation of miR-1 expression, elevation of HDAC4 protein levels, and decreases in the expression of cardiac myosin-related transcription factors (MRTF) A and B in the lateral femoral muscle. This miR-1 downregulation increases the HDAC4 expression, which in turn inhibits the myosin heavy chain-I (MyHC-I), a regulator of myosin MEF2 and SRF activity, and MRTF/SRF activity correlates with the regulation of MyHC-I expression (Allen et al., 2005). The decrease in MRTF/SRF and miR-1 expression accounts for significant decreases in the proportions of type I fibers in patients with severe COPD, altering the quadriceps fiber types as a result. In addition, the activity of MEF2 and SRF is reduced in COPD, thus downregulating the miR-1 expression (Lewis et al., 2012). Decreases in miR-1 has also been shown to inhibit the calcium-regulated neuro phosphatase (CaN) pathway (Ikeda et al., 2009). Inhibiting this pathway also decreases the activation of the essential muscle-related transcription factor MEF2 (Wu et al., 2001), indicating a shift from type I to type II fiber types and muscle atrophy in patients with severe COPD(Wu et al., 2001; Lewis et al., 2012). It has also been found that miR-1 regulates the nerves associated with muscle required for force production (Anderson et al., 2006). As discussed in the experiments of Puig et al., miR-1 expression is reduced in the diaphragm for mild, moderate, and severe COPD cases, a pattern of expression that correlates with sustained loading of the diaphragm. In an experimental model of overload-induced muscle hypertrophy proposed by McCarthy et al., (McCarthy and Esser, 2007),miR-1 expression is also downregulated in muscles that are subject to overload, suggesting that miR-1 downregulation could cause muscle adaptation to overload through the elimination of the expression of gene transcripts and pathways associated with muscle growth.

**FIGURE 1:**
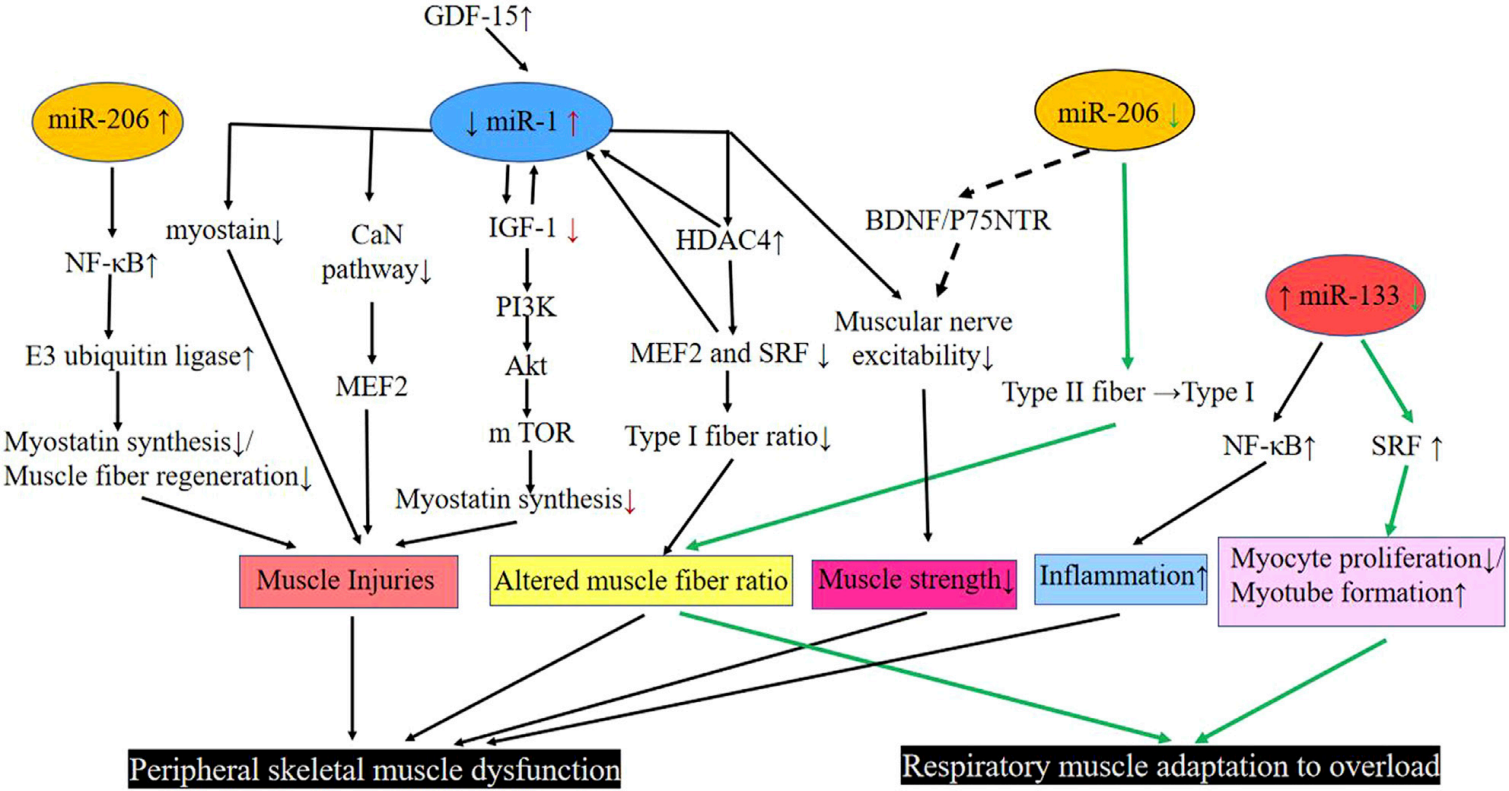
Pathways involved in miR-1, −206, and −33 regulation of respiratory muscle and peripheral skeletal muscle dysfunction in COPD. Black line: Peripheral skeletal muscle dysfunction; Green line: Respiratory muscle adaptation to overload. Abbreviations: Akt, protein kinase B; BDNF, brain-derived neurotrophic factor; CaN pathway, calcium-regulated neurophosphatase pathway; COPD, chronic obstructive pulmonary disease; GDF-15, growth differentiation factor 15; HDAC4, histone deacetylase 4; IGF-1, insulin-like growth factor-1; m TOR, mammalian target of rapamycin; Myostatin, muscle growth inhibitor; MEF2, muscle enhancement factor 2; NF-κB, enhanced κ-light chain of nuclear factor-activated B cells; P75NTR, neurotrophic factor receptor; PI3K, phosphatidylinositol 3 kinase; SRF, serum response factor; down (↓); up (↑); transform (→).

In summary (Figure 1), skeletal muscle miR-1 reduces the rate of protein synthesis by regulating the expression of IGF-1 in the IGF-1/PI3K/Akt/m TOR pathway. It increases the activity of the myogenic inhibitor pathway and promotes skeletal muscle atrophy by interacting with GDF-15. Downregulated miR-1 increases the expression of HDAC4, which in turn inhibits the activity of MEF2 and SRF, reducing the proportion of muscle type I fibers. miR-1 further inhibits MEF2 by suppressing the CaN pathway, thus leading to an abnormal muscle fiber type shift. In addition, it regulates the nerves that innervate the muscle and regulates the diaphragm’s adaptation to overload.

### 4.2 miR-206

#### 4.2.1 miR-206 affects skeletal muscle protein degradation

MiR-206 is expressed in skeletal muscles and functions as a component of myogenesis in humans and mice (Kim et al., 2006; Koutsoulidou et al., 2011). Elevated miR-206 in the plasma of COPD patients has been found to be positively correlated with enhanced p50 (NF-κB p50) of κ-light chain of nuclear factor-activated B cells in the quadriceps muscle of patients with GOLD I and II COPD, leading to skeletal muscle dysfunction in COPD (Donaldson et al., 2013). An increase in expression of the miR-206 and NF-κB, an important signaling pathway involved in skeletal muscle mass depletion, has been observed in the quadriceps muscles of severe COPD patients. Furthermore, NF-κB activation can act on E3 ubiquitin ligase to activate the protease hydrolysis system, leading to the degradation of specific muscle proteins that prevents regeneration of myogenic fibers and massive loss of muscle mass. Collectively, these lead to skeletal muscle dysfunction (McCarthy and Esser, 2007; McCarthy et al., 2009; Puig-Vilanova et al., 2015) (Figure 1).

#### 4.2.2 miR-206 affects the diaphragm fibre type and muscle nerve excitability

Previous experiments have found that deletion of the miR-206 gene delays the regeneration of the tibialis anterior muscle in mice that are exposed to cardiotoxin injury. These mice also show smaller muscle fiber cross-sections compared to wild-type control mice (Liu et al., 2012). In addition, downregulation of the expression of myomiRs, especially the miR-206, causes severe damage, fiber type changes and degeneration of diaphragm fibers with calcium deposition, mineralization, and fibrosis, suggesting a close relationship between the miR-206 expression level and diaphragm function (Liu et al., 2012). In the aforementioned experiment by Puig et al., expression of miR-206 was lower than the miR-1 and miR-133, and was detected both in moderate and severe COPD diaphragm muscles that shifted from type II to type I fiber types; this change was attributed to the downregulation of miR-206 expression (Puig-Vilanova et al., 2014a) (Figure 1).In order to ensure proper skeletal muscle function, there is an associated innervation of skeletal muscle, during this period, skeletal muscle tissue and axons synergize to form neuromuscular connections that allow the muscle to function. It has been found that the upregulated miR-206 promotes intercommunication between the muscle fibers and motor neuron axons by targeting various molecules such as brain-derived neurotrophic factor (BDNF), neurotrophic factor receptor (p75NTR) and HDAC4 (Williams et al., 2009; Miura et al., 2012). Therefore, miR-206, which is aberrantly expressed in COPD patients, may also regulate muscle fiber innervation through this pathway (Anderson et al., 2006; Jeng et al., 2009; Barreiro and Sznajder, 2013) (Figure 1).

### 4.3 miR-133 regulates diaphragm adaptation to overload and peripheral skeletal muscle inflammation

Studies generally agree that overexpressed miR-133 inhibits myotube formation by suppressing the SRF-stimulated myogenic cell proliferation (Chen et al., 2006). As previously described, miR-133 expression was decreased in the diaphragm (Puig-Vilanova et al., 2014a). miR-133 expression was also downregulated in experimental models of muscle hypertrophy in muscles subjected to overload, and SRF expression was elevated, inhibiting myoblast proliferation and promoting myotube formation, suggesting that downregulated miR-133 promotes the adaptive effects of muscle to overload (McCarthy and Esser, 2007). Diaphragm is overloaded due to airflow limitation and continuous overinflation in COPD patients (Ottenheijm et al., 2008; Klimathianaki et al., 2011). Therefore, miR-133 expression was downregulated in COPD diaphragm and may reduce the overproliferation of diaphragm cells by increasing the expression of SRF. In this way, the oxygen and energy consumption of diaphragm cells may be reduced, thus regulating the adaptation of the diaphragm to overload (Figure 1). Research has further indicated that elevation in plasma miR-133 is positively correlated with nuclear NF-κB p50 in the quadriceps muscle of patients with GOLD I and stage II COPD, and is positively correlated with tumor necrosis factor-α, interleukin 2, and interleukin 5 levels, which may be associated with the inflammatory response in the quadriceps muscle (Donaldson et al., 2013) (Figure 1).

### 4.4 Other myomiRs

In addition to the studied myomiRs discussed above, miR-499 and miR-208b have been also thoroughly investigated in the context of COPD. It has been shown that miR-499 and miR-208b are encoded slow myosin heavy chain gene, so they are only expressed in type I fibers (van Rooij et al., 2009). Moreover, knockdown of the genes encoding miR-499 and miR-208b decreased the proportion of type I fibers, while when expression of miR-499 and miR-208b was restored, the proportion of type I fibers and endurance in mice did not differ from healthy mice (McCarthy et al., 2009; van Rooij et al., 2009; Güller and Russell, 2010).In the quadriceps muscle of patients with severe COPD as type I fibers decrease miR-499 and miR-208b expression decreases (van Rooij et al., 2009; Güller and Russell, 2010). Whittom et al. (1998) previously found that the percentage of type I decreased by roughly 20% in severe COPD patients, while type II fibers increased by 10%; the remaining type I fibers were catabolized into the blood. Therefore, miRNAs enter the blood with the degradation of type I fibers and consequently increase the level of miRNAs in the blood (Donaldson et al., 2013). As a result, the decreased expression of miR-499 and miR-208b in plasma suggest a decrease in the proportion of skeletal muscle type I fibers in COPD patients, implicating a more severe disease. Recent work has demonstrated that the transition from type I to type II fibers is associated with increased mortality rates (Patel et al., 2014). Additionally, elevated plasma miR-499 in COPD patients is positively correlated with NF-κB p50 in the quadriceps muscle; NF-κB is further associated with inflammatory responses, implicating the role of miR-499 in regulating quadricep inflammation (Attaix et al., 2005; Donaldson et al., 2013).

## 5 Conclusion

In conclusion, skeletal muscle dysfunction in COPD is manifested in the transformation of respiratory and peripheral skeletal muscle fiber types and the reduction of muscle cross-sectional area, muscle strength, endurance, and fatigue resistance. Furthermore, respiratory and peripheral muscle fiber type transformation, cross-sectional area size, and myostatin synthesis and degradation are all influenced by myomiR expression. The myomiR expression is also regulated by muscle-specific transcription factors, such as HDAC4, MEF2, IGF-1, and SRF. The myomiRs are expressed differently in respiratory and peripheral skeletal muscles of COPD patients, as well as in patients with different degrees of disease severity. The former may be related to continuous muscle loading, while the latter may be related to the integrity of body composition and the interaction of various muscle growth factors.Furthermore, different myomiRs play regulatory roles in skeletal muscle function in COPD patients. The miR-1, miR-499 and miR-208b are focused on regulating the changes in skeletal muscle fiber type. In addition, miR-1 and miR-206 are associated with muscle protein synthesis and degradation; miR-1 and miR-133 have been demonstrated to play roles in regulating diaphragm adaptation to overload. Moreover, miR-206, miR-133 and miR-499 have been found to be associated with NF-κB p50 in the quadriceps muscle, while circulating miR-206 may potentially be used as a biomarker to assess skeletal muscle dysfunction in COPD patients.

Collectively, the investigations to date regarding the role and mechanism of myomiRs in skeletal muscle dysfunction in COPD may provide future avenues for exploration for the prevention and treatment of skeletal muscle dysfunction in COPD.
